# Design and Implementation of Vector Tracking Loop for High-Dynamic GNSS Receiver

**DOI:** 10.3390/s21165629

**Published:** 2021-08-20

**Authors:** Rongjun Mu, Teng Long

**Affiliations:** School of Astronautics, Harbin Institute of Technology, Harbin 150001, China; murjun@163.com

**Keywords:** GNSS, vector tracking, high-dynamic, hardware receiver

## Abstract

For the tracking of high-dynamic satellite navigation signals, the conventional scalar tracking loop (STL) is vulnerable. Frequent signal-tracking interruption affects the continuity of navigation. The vector tracking loop (VTL) can overcome this disadvantage. However, there are some difficulties in implementing existing vector tracking methods on a real-time hardware receiver, such as the synchronization problem and computation load. This paper proposes an implementation framework of VTL based on a partial open-loop numerically controlled oscillator (NCO) control mode that can be implemented with minor modifications on an existing receiver platform. The structure of VTL, the design of the navigation filter, and the key points of hardware implementation are introduced in detail. Lastly, the VTL performance was verified by a GPS simulator test. The results show that the proposed VTL can run in real-time and be significantly improved in the tracking continuity of high-dynamic signals, tracking sensitivity, positioning accuracy, and recovery time for interrupted signals compared with those of STL.

## 1. Introduction

Global navigation satellite systems (GNSS) are widely used in positioning and navigation tasks for artificial flight vehicles as a high-availability, high-precision, and low-cost positioning technology. Some of these vehicles have high velocity and acceleration, such as missiles, rockets, and hypersonic aircraft. This requires the GNSS receiver to have the ability to track high-dynamic signals. As early as 1988, the Jet Propulsion Laboratory (JPL) researched high-dynamic global positioning system (GPS) signal tracking according to the application requirement of the GPS Range Application Joint Program Office (RAJPO) [1]. In that report, a scenario with an acceleration of 50 g and a jerk of 100 g/s was proposed as the upper bound for the study of high-dynamic signal tracking.

There are three main factors affecting the high-dynamic performance of a receiver: Loop filter characteristics, oscillator noise, and oscillator vibration sensitivity [2]. The latter two can be improved on the hardware-design level [3], but the first needs to be improved on the software and algorithmic levels, which are the focus of this paper. There are many proposed methods on how to achieve high-dynamic signal tracking, such as second-order frequency lock loop (FLL) assisted third-order phase lock loop (PLL) [4], Kalman filter-based [5], maximum likelihood-based [6], and fractional Fourier transform-based [7,8]. However, these methods focus only on a single channel and ignore overall performance. Subject to the nature of scalar tracking loop (STL) in which signal-tracking channels are independent of each other, the optimization of one channel cannot optimize the receiver. Some channels may still lose the lock when a high-dynamic or weak signal is encountered. The problem of the continuity of signal tracking has not been solved. Fortunately, the vector tracking loop (VTL) was proposed to overcome the disadvantage of STL [9]. The principle of VTL is to combine the signal tracking of all channels via the receiver’s position, velocity, and time (PVT) solution, which gives several advantages. First, information fusion between channels can reduce noise and keep the error of the local replica signal small enough to stay within the linear region of tracking [10]. Second, strong signals can assist in weak-signal tracking, and low-dynamic signals can assist in high-dynamic-signal tracking. Third, when parts of signals are interrupted, a VTL can bridge these signals through the PVT solution [11]. In theory, vector tracking is an ideal framework for designing a satellite navigation receiver.

**Figure 1:**
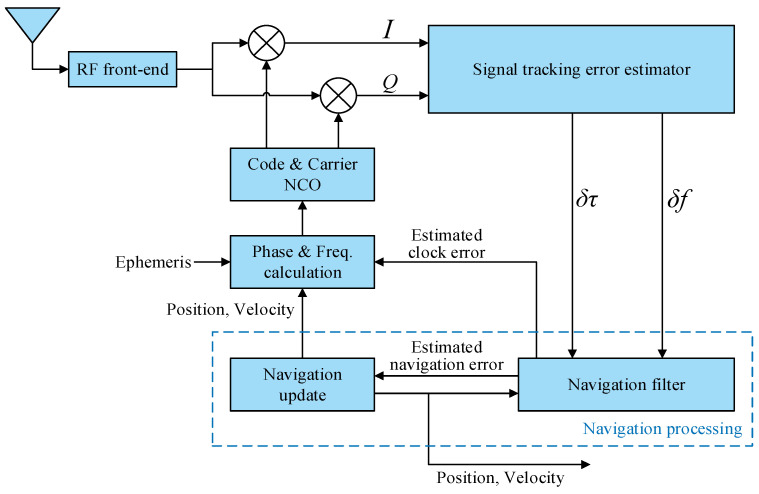
The unified framework of vector tracking loop.

There are two types of VTL, centralized and cascaded. The centralized VTL is rarely used on account of its computational complexity and nonlinearity [12]. The cascaded VTL, which is widely studied, can be summarized into a unified framework [13], as shown in Figure 1. The basic principle of this framework is that the code phase and carrier frequency of each channel, which is used to control code and carrier numerically controlled oscillator (NCO), are calculated by the receiver’s PVT solution. The PVT solution is corrected by the navigation errors that are estimated by the navigation filter according to the error of the code phase and the carrier frequency, which are estimated by the signal tracking error estimator. Within the unified framework, the difference between various vector tracking methods is implementing the signal tracking error estimators, such as discriminator-based [14] and prefilter-based [10,15] estimators. Most researchers implement a VTL using a software-defined GNSS receiver (SDGR) [16,17,18]. In SDGR, a digital intermediate-frequency (IF) signal is collected and then batch processed in a general-purpose processor (GPP) or a digital signal processor (DSP). Signal processing is flexible, so that the code phase and carrier phase of the local replica signal can be freely controlled. The unified framework is easy to implement in an SDGR. However, in a GNSS hardware receiver, NCO and correlator run at high speed in a special integrated circuit or a field-programmable gate array (FPGA), so it is hard to accurately control the code phase at a certain moment. Therefore, the framework needs to be modified to fit hardware implementation. An implementation framework of VTL based on a partial open-loop NCO control mode is proposed in this paper. In this framework, the VTL can easily be implemented using the existing correlator and NCO control interface, and it has the ability of high-dynamic signal tracking.

The remainder of the paper is organized as follows. Section 2 introduces our methods, including the structure and principle of the proposed VTL implementation framework, the method of navigation processing, a processing strategy of weak-signal channels, and the structure and key design points of the vector tracking hardware receiver. In Section 3, the results of high-dynamic experiments based on a GPS simulator are analyzed. Section 4 discusses some future research directions of VTL. Section 5 summarizes the study.

## 2. Methods

### 2.1. Vector Tracking Loop

#### 2.1.1. Loop Structure

**Figure 2:**
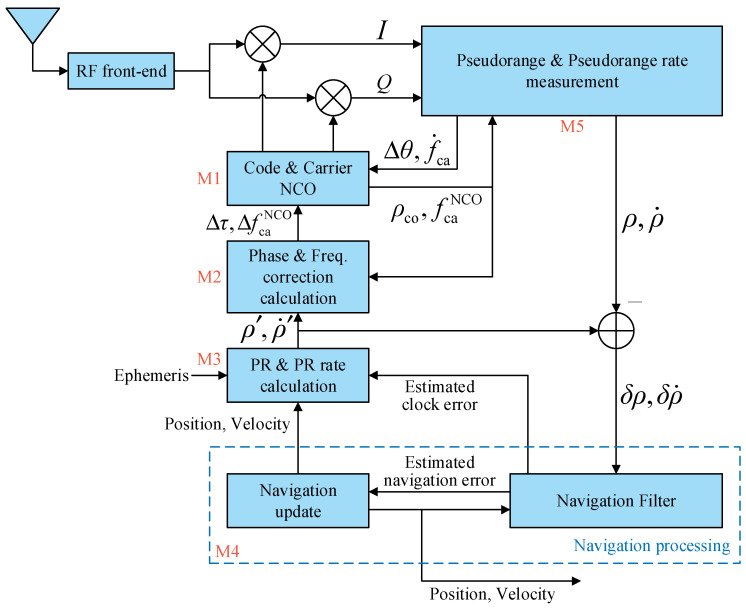
Implementation framework of vector tracking loop.

The diagram of the proposed implementation framework of VTL based on a partial open-loop NCO control mode is shown in Figure 2.

Symbols in Figure 2:

M1–M5 the module number, which will be referenced below;

$\rho_{\mathrm{co}}$ code pseudorange, which is calculated by the code phase;

$f_{\mathrm{ca}}^{\mathrm{NCO}}$ carrier NCO frequency;

${\dot{f}}_{\mathrm{ca}}$ estimated carrier frequency change rate;

Δτ code phase correction value;

$\Delta f_{\mathrm{ca}}^{\mathrm{NCO}}$ carrier NCO frequency correction value;

Δθ carrier phase correction value;

$\rho ,\dot{\rho }$ measured pseudorange and pseudorange rate;

$\rho^{\prime },{\dot{\rho }}^{\prime }$ calculated pseudorange and pseudorange rate;

$\delta \rho ,\delta \dot{\rho }$ pseudorange error and pseudorange rate error.

Code and carrier NCO (M1) generates local replica code and carrier that are phase-aligned with the real signal. In the unified framework of VTL, NCOs run in full closed-loop mode; this means that each NCO control command is computed by the PVT solution. The term “partial open-loop” refers to NCOs running in open-loop mode during the navigation update interval, and then being corrected after the navigation update. During open-loop tracking, the NCO control command does not come from the PVT solution, but from the channel’s own information. The open-loop NCO control command requires high accuracy, that is, the open-loop error being less than the closed-loop error. To achieve this, carrier NCO frequency $f_{\mathrm{ca}}^{\mathrm{NCO}}$ is used to calculate code NCO frequency $f_{\mathrm{co}}^{\mathrm{NCO}}$. Because of the strict linear relationship between the carrier frequency and code frequency, and code frequency being much less than radio-frequency (RF) carrier frequency, the calculated code frequency is so accurate that the code phase drift is very small in the short term. The calculation formula of $f_{\mathrm{co}}^{\mathrm{NCO}}$ is

(1) $$f_{\mathrm{co}}^{\mathrm{NCO}}=\left(1+\frac{f_{\mathrm{ca}}^{\mathrm{NCO}}-f_{\mathrm{IF}}}{F_{\mathrm{RF}}}\right)F_{\mathrm{co}}$$

where $f_{\mathrm{IF}}$ is the IF frequency after down-conversion, $F_{\mathrm{RF}}$ is the RF carrier center frequency, $F_{\mathrm{co}}$ is the nominal code frequency (e.g., GPS L1 C/A signal $F_{\mathrm{RF}}$ = 1575.42 MHz, $F_{\mathrm{co}}$ = 1.023 MHz). For carrier open-loop tracking, carrier frequency change rate ${\dot{f}}_{\mathrm{ca}}$ is used to control carrier NCO frequency update so as to track the change in carrier doppler caused by the vehicle motion. ${\dot{f}}_{\mathrm{ca}}$ comes from the pseudorange rate measurement module (M5); this is explained in detail in Section 2.1.2. The update process of $f_{\mathrm{ca}}^{\mathrm{NCO}}$ is

(2) $$f_{\mathrm{ca}}^{\mathrm{NCO}+}=f_{\mathrm{ca}}^{\mathrm{NCO}-}+{\dot{f}}_{\mathrm{ca}}T_{\mathrm{NCO}}$$

where the superscript − denotes before update, superscript + denotes after update, $T_{\mathrm{NCO}}$ is the carrier NCO update period.

Correction information comes from the optimal estimation of the PVT solution by the navigation processing module (M4). Since the NCO is not directly controlled by the PVT solution during open-loop tracking, the measurement extracted from the channel is not tracking error but pseudorange and pseudorange rate (M5). The code pseudorange and carrier NCO frequency represent the current state of open-loop tracking, the pseudorange and pseudorange rate calculated by the estimated PVT solution (M3) are the expected state of signal tracking, the difference between the current and expected state results in the code phase correction value Δτ and carrier NCO frequency correction value $\Delta f_{\mathrm{ca}}^{\mathrm{NCO}}$ (M2):

(3) $$\Delta \tau =-\frac{\rho^{\prime }-\rho_{\mathrm{co}}}{\lambda_{\mathrm{co}}}$$

(4) $$\Delta f_{\mathrm{ca}}^{\mathrm{NCO}}=-\frac{{\dot{\rho }}^{\prime }}{\lambda_{\mathrm{ca}}}-\left(f_{\mathrm{ca}}^{\mathrm{NCO}}-f_{\mathrm{IF}}\right)$$

where $\lambda_{\mathrm{co}}$ is the code length, $\lambda_{\mathrm{ca}}$ is the RF wavelength. Due to the influence of the receiver clock drift, the relationship between the frequency of the digital signal after A/D sampling and the real signal is

(5) $$f_{\mathrm{real}}=\left(1+df_{r}\right)f_{\mathrm{digital}}$$

where $df_{r}$ is the receiver clock drift, its unit is usually ppm. Therefore, more accurately, Equation (4) should be written as

(6) $$\Delta f_{\mathrm{ca}}^{\mathrm{NCO}}=-\frac{{\dot{\rho }}^{\prime }}{\lambda_{\mathrm{ca}}\left(1+df_{r}\right)}-\left(f_{\mathrm{ca}}^{\mathrm{NCO}}-f_{\mathrm{IF}}\right)$$

In partial open-loop NCO control mode, the tracking of each channel is independent during open-loop tracking, and the correction process couples all channels together to achieve vector tracking. Because the correction value is the increment in the current internal state of the NCO, it does not care about the absolute value of the code phase and carrier NCO frequency, there is no special requirement for the time to perform these corrections.

#### 2.1.2. Pseudorange and Pseudorange Rate Measurement

**Figure 3:**
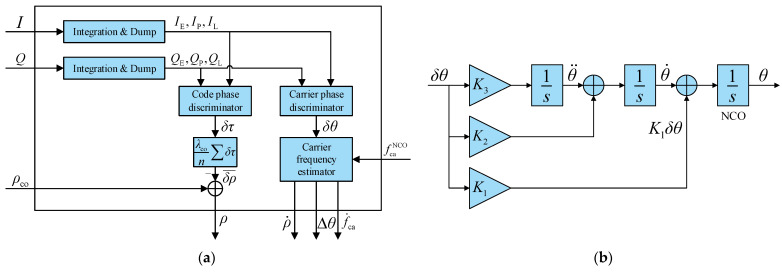
(**a**) Diagram of pseudorange and pseudorange rate measurement; (**b**) structure of the third-order loop filter.

The principle of pseudorange and pseudorange rate measurement is shown in Figure 3a.

The code phase discriminator outputs code phase error δτ between the local and actual code phase, so measured pseudorange is obtained by

(7) $$\rho =\rho_{\mathrm{co}}-\lambda_{\mathrm{co}}\delta \tau$$

In general, there are several code phase discriminator outputs in a pseudorange measurement period. Because of the high accuracy of code open-loop tracking, the code phase error can be unchanged during this period. Taking the average of these code phase errors can reduce the pseudorange measurement noise:

(8) $$\rho =\rho_{\mathrm{co}}-\lambda_{\mathrm{co}}\frac{1}{n}\sum_{i=1}^{n}\delta \tau_{i}$$

The function of the carrier frequency estimator in Figure 3a is to calculate carrier frequency $f_{\mathrm{ca}}$, pseudorange rate $\dot{\rho }$, carrier phase correction value Δθ, and carrier frequency change rate ${\dot{f}}_{\mathrm{ca}}$ from carrier phase error δθ and carrier NCO frequency $f_{\mathrm{ca}}^{\mathrm{NCO}}$. The calculation formulas of the carrier frequency estimator can be derived from alpha-beta-gamma filter. The state variables of the filter are carrier phase error δθ, carrier frequency error $\delta f_{\mathrm{ca}}$, and carrier frequency change rate error $\delta {\dot{f}}_{\mathrm{ca}}$. The measurement of the filter is δθ. The filtering equation is as follows:

(9) $$\left[\begin{array}{c} \delta {\hat{\theta }}_{k}\\ \delta {\hat{f}}_{\mathrm{ca},k}\\ \delta {\hat{\dot{f}}}_{\mathrm{ca},k} \end{array}\right]=\left[\begin{array}{ccc} 1 & T_{\mathrm{coh}} & 0.5T_{\mathrm{coh}}^{2}\\ 0 & 1 & T_{\mathrm{coh}}\\ 0 & 0 & 1 \end{array}\right]\left[\begin{array}{c} \delta {\hat{\theta }}_{k-1}\\ \delta {\hat{f}}_{\mathrm{ca},k-1}\\ \delta {\hat{\dot{f}}}_{\mathrm{ca},k-1} \end{array}\right]+\left[\begin{array}{c} \alpha \\ \beta \\ \gamma \end{array}\right]\left(\delta \theta -\delta {\hat{\theta }}_{k-1}-T_{\mathrm{coh}}\delta {\hat{f}}_{\mathrm{ca},k-1}-0.5T_{\mathrm{coh}}^{2}\delta {\hat{\dot{f}}}_{\mathrm{ca},k-1}\right)$$

where the caret symbol (^) denotes the estimated value, $T_{\mathrm{coh}}$ is the coherent integration time, k denotes the update epoch. α, β, and γ are parameters of the alpha-beta-gamma filter. From the conventional point of view, these three errors should be corrected after each filter update, and then $\delta {\hat{\theta }}_{k-1}$,$\delta {\hat{f}}_{\mathrm{ca},k-1}$, and $\delta {\hat{\dot{f}}}_{\mathrm{ca},k-1}$ are all equal to zero. However, according to Equation (6), the correction effect of pseudorange rate to carrier NCO frequency is equivalent to an additional carrier frequency error for this filter. So, $\delta {\hat{f}}_{\mathrm{ca},k-1}$ may not be equal to zero. According to the definition of carrier frequency error, $\delta {\hat{f}}_{\mathrm{ca}}$ can be replaced by $f_{\mathrm{ca}}-f_{\mathrm{ca}}^{\mathrm{NCO}}$, where $f_{\mathrm{ca}}$ is the estimated carrier frequency. Then Equation (9) transforms to

(10) $$\left[\begin{array}{c} \delta {\hat{\theta }}_{k}\\ f_{\mathrm{ca},k}-f_{\mathrm{ca}}^{\mathrm{NCO}}\\ \delta {\hat{\dot{f}}}_{\mathrm{ca},k} \end{array}\right]=\left[\begin{array}{ccc} 1 & T_{\mathrm{coh}} & 0.5T_{\mathrm{coh}}^{2}\\ 0 & 1 & T_{\mathrm{coh}}\\ 0 & 0 & 1 \end{array}\right]\left[\begin{array}{c} 0\\ f_{\mathrm{ca},k-1}-f_{\mathrm{ca}}^{\mathrm{NCO}}\\ 0 \end{array}\right]+\left[\begin{array}{c} \alpha \\ \beta \\ \gamma \end{array}\right]\left[\delta \theta -T_{\mathrm{coh}}\left(f_{\mathrm{ca},k-1}-f_{\mathrm{ca}}^{\mathrm{NCO}}\right)\right]$$

By organizing Equation (10) and simplifying some symbols, update equations of the carrier frequency estimator can be obtained:

(11) $$\Delta f_{\mathrm{ca}}=f_{\mathrm{ca}}^{\mathrm{NCO}}-f_{\mathrm{ca}}^{-}$$

(12) $$\delta \theta_{0}=\delta \theta +\Delta f_{\mathrm{ca}}T_{\mathrm{coh}}$$

(13) $$\Delta \theta =\alpha \cdot \delta \theta_{0}-\Delta f_{\mathrm{ca}}T_{\mathrm{coh}}$$

(14) $$f_{\mathrm{ca}}^{+}=f_{\mathrm{ca}}^{-}+\beta \cdot \delta \theta_{0}$$

(15) $${\dot{f}}_{\mathrm{ca}}^{+}={\dot{f}}_{\mathrm{ca}}^{-}+\gamma \cdot \delta \theta_{0}$$

Essentially, the carrier frequency estimator is a digital third-order PLL. The structure of the third-order loop filter is shown in Figure 3b. $\Delta f_{\mathrm{ca}}T_{\mathrm{coh}}$ is the additional carrier phase error caused by the difference between the NCO frequency and the estimated frequency. $\delta \theta_{0}$ is the carrier phase error input to the PLL. Because the effect of $\Delta f_{\mathrm{ca}}T_{\mathrm{coh}}$ has been removed from the directly measured carrier phase error, the estimation result of $f_{\mathrm{ca}}$ does not change even if $f_{\mathrm{ca}}^{\mathrm{NCO}}$ is corrected externally. This is the basis of the vector carrier tracking that the carrier frequency estimation is independent of the carrier NCO control. Δθ is used to accomplish carrier phase locking. ${\dot{f}}_{\mathrm{ca}}$ is provided to the carrier NCO for open-loop update. The value of α,β,γ can be determined by typical parameters of third-order PLL [19], as follows:

(16) $$\{\begin{array}{l} \alpha =K_{1}T_{\mathrm{coh}}=2.4\omega_{0}T_{\mathrm{coh}}\\ \beta =K_{2}T_{\mathrm{coh}}=1.1\omega_{0}^{2}T_{\mathrm{coh}}\\ \gamma =K_{3}T_{\mathrm{coh}}=\omega_{0}^{3}T_{\mathrm{coh}} \end{array}$$

(17) $$\omega_{0}=1.275B_{n}$$

where $B_{n}$ is the equivalent noise bandwidth, and $\omega_{0}$ is the natural frequency.

**Figure 4:**
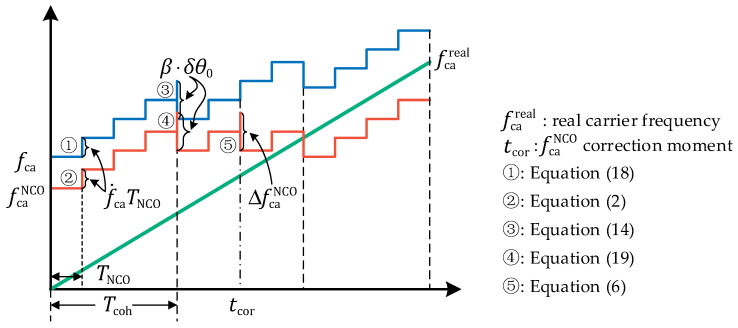
Diagram of the update process of $f_{\mathrm{ca}}$ and $f_{\mathrm{ca}}^{\mathrm{NCO}}$. (For more clearly, a coherent integration time contains four carrier NCO update periods.).

Figure 4 shows the update process of $f_{\mathrm{ca}}$ and $f_{\mathrm{ca}}^{\mathrm{NCO}}$ more visually. Note that $f_{\mathrm{ca}}$ needs to be updated synchronously with $f_{\mathrm{ca}}^{\mathrm{NCO}}$ after each carrier NCO update:

(18) $$f_{\mathrm{ca}}^{+}=f_{\mathrm{ca}}^{-}+{\dot{f}}_{\mathrm{ca}}T_{\mathrm{NCO}}$$

And $f_{\mathrm{ca}}^{\mathrm{NCO}}$ needs to increase the same increment with $f_{\mathrm{ca}}$ when the carrier frequency estimator update:

(19) $$f_{\mathrm{ca}}^{\mathrm{NCO}+}=f_{\mathrm{ca}}^{\mathrm{NCO}-}+\beta \cdot \delta \theta_{0}$$

After carrier frequency estimating, measured pseudorange rate is obtained by

(20) $$\dot{\rho }=-\left(1+df_{r}\right)\left(f_{\mathrm{ca}}-f_{\mathrm{IF}}\right)\lambda_{\mathrm{ca}}$$

### 2.2. Navigation Processing

Navigation processing comprises two parts: Navigation update and navigation filter. The function of the navigation update is to predict the current navigation state according to the last navigation state. The function of the navigation filter is to estimate navigation errors according to pseudorange and pseudorange rate measurements.

#### 2.2.1. Navigation Update

For high-dynamic scenarios, the position, velocity, and acceleration are used as the navigation state. Navigation update is based on the constant acceleration criterion, assuming constant acceleration within the update interval. The navigation update process is as follows:

(21) $$\hat{L}=L^{-}+\frac{v_{N}^{-}}{R_{M}+h^{-}}T_{\mathrm{nav}}$$

(22) $$\hat{\lambda }=\lambda^{-}+\frac{v_{E}^{-}}{\left(R_{N}+h^{-}\right)\cos L^{-}}T_{\mathrm{nav}}$$

(23) $$\hat{h}=h^{-}-v_{D}^{-}T_{\mathrm{nav}}$$

(24) $${\;\hat{v}}^{n}=v^{n-}+a^{n-}T_{\mathrm{nav}}$$

(25) $${\;\hat{a}}^{n}=a^{n-}$$

(26) $${\hat{b}}_{c}=b_{c}^{-}+d_{c}^{-}T_{\mathrm{nav}}$$

(27) $${\hat{d}}_{c}=d_{c}^{-}$$

Symbols in Equations (21)–(27):

L,λ,h latitude, longitude, and altitude;

$v^{n}$$\left[v_{E},v_{N},v_{D}\right]$, velocity vector in the geographic frame;

$a^{n}$$\left[a_{E},a_{N},a_{D}\right]$, acceleration vector in the geographic frame;

$b_{c}$ equivalent range error of the receiver clock offset;

$d_{c}$ equivalent velocity error of the receiver clock drift;

$R_{M},R_{N}$ curvature radiuses of the Earth in the meridian and prime vertical directions;

$T_{\mathrm{nav}}$ navigation update period.

#### 2.2.2. Navigation Filter

The errors of the navigation state are selected as state variables; the state vector is expressed as

(28) $$X={\left[\delta p^{n},\delta v^{n},\delta a^{n},\delta b_{c},\delta d_{c}\right]}^{T}$$

The state equation is

(29) $$\dot{X}=\left[\begin{array}{ccccc} 0 & I & 0 & 0 & 0\\ 0 & 0 & I & 0 & 0\\ 0 & 0 & 0 & 0 & 0\\ 0 & 0 & 0 & 0 & 1\\ 0 & 0 & 0 & 0 & 0 \end{array}\right]X$$

The measurement equation is

(30) $$Z=\left[\begin{array}{c} \delta \rho \\ \delta \dot{\rho } \end{array}\right]=\left[\begin{array}{c} \rho^{\prime }-\rho \\ {\dot{\rho }}^{\prime }-\dot{\rho } \end{array}\right]=\left[\begin{array}{ccccc} e^{n} & 0 & 0 & -1 & 0\\ 0 & e^{n} & 0 & 0 & -1 \end{array}\right]X$$

where $e^{n}$ is the line-of-sight (LOS) unit vector from satellite to receiver. The satellite clock error and various errors in the signal propagation process [20] should be considered when calculating $\rho^{\prime }$ and ${\dot{\rho }}^{\prime }$:

(31) $$\rho^{\prime }=\rho_{r}^{s}+c\left(dt_{r}-dt_{s}-dt_{\mathrm{rel}}+dt_{\mathrm{GD}}+dt_{\mathrm{Sagnac}}+dt_{\mathrm{ion}}+dt_{\mathrm{tro}}\right)$$

(32) $${\dot{\rho }}^{\prime }={\dot{\rho }}_{r}^{s}+c\left(df_{r}-\delta f_{s}-\delta f_{\mathrm{rel}}+\delta f_{\mathrm{Sagnac}}\right)$$

Symbols in Equations (31) and (32):

$\rho_{r}^{s}\;$ satellite-receiver geometric range;

$dt_{s}$ satellite clock offset;

$dt_{\mathrm{rel}}$ relativistic correction;

$dt_{\mathrm{GD}}$ group delay;

$dt_{\mathrm{Sagnac}}$ Sagnac delay;

$dt_{\mathrm{ion}}$ ionosphere delay;

$dt_{\mathrm{tro}}$ tropospheric delay;

${\dot{\rho }}_{r}^{s}\;$ satellite-receiver relative velocity;

$df_{s}$ satellite clock drift;

$df_{\mathrm{rel}}$ relativistic correction rate;

$df_{\mathrm{Sagnac}}$ Sagnac delay rate;

c speed of light.

To make the navigation filter more robust in the case of unstable measurement noise, a Huber-based Kalman filter is introduced [21,22]. The filtering process is given in Algorithm 1.

**Algorithm 1.** Huber-based Kalman filter for the navigation filterStep 1Calculate one-step prediction variance matrix: $P_{k/k-1}=\Phi_{k/k-1}P_{k-1}\Phi_{k/k-1}^{T}+Q_{k-1}$.Step 2Calculate initial state vector: $K_{k}=P_{k/k-1}H_{k}^{T}{\left(H_{k}P_{k/k-1}H_{k}^{T}+R_{k}\right)}^{-1}$, ${\hat{X}}_{k}^{0}=K_{k}Z_{k}$.Step 3Calculate residual vector: $\xi_{X}=P_{k/k-1}^{-1/2}\left({\hat{X}}_{k}^{j}-{\hat{X}}_{k/k-1}\right)$, $\xi_{Z}=R_{k}^{-1/2}\left(H_{k}{\hat{X}}_{k}^{j}-Z_{k}\right)$. j is the number of iterations, and its initial value is 0.Step 4Calculate Huber weight matrix: $\Psi_{\xi ,X}=diag\left[ℋ\left(\xi_{X}\right)\right]$, $\Psi_{\xi ,Z}=diag\left[ℋ\left(\xi_{Z}\right)\right]$. ℋ is Huber function, $ℋ\left(\xi \right)=\{\begin{array}{l} 1,\left|\xi \right|<\gamma \\ \frac{\gamma }{\xi },\left|\xi \right|\geq \gamma \end{array}$, γ is a parameter.Step 5Calculate $P_{k/k-1}^{\prime }$ and $R_{k}^{\prime }$:$P_{k/k-1}^{\prime }=P_{k/k-1}^{1/2}\Psi_{\xi ,X}^{-1}{\left(P_{k/k-1}^{1/2}\right)}^{T}$, $R_{k}^{\prime }=R_{k}^{1/2}\Psi_{\xi ,Z}^{-1}{\left(R_{k}^{1/2}\right)}^{T}$.Step 6Calculate state vector: $K_{k}=P_{k/k-1}^{\prime }H_{k}^{T}{\left(H_{k}P_{k/k-1}^{\prime }H_{k}^{T}+R_{k}^{\prime }\right)}^{-1}$, ${\hat{X}}_{k}^{j+1}=K_{k}Z_{k}$.Step 7Check if $\left\|X_{k}^{j+1}-X_{k}^{j}\right\|$ is larger than a threshold. If yes, go back to Step 3.Step 8Calculate state vector error variance matrix: $P_{k}=\left(I-K_{k}H_{k}\right)P_{k/k-1}^{\prime }$.

In Steps 5, $P_{k/k-1}^{1/2}$ and $R_{k}^{1/2}$ are lower triangular matrixes of the Cholesky factorization of $P_{k/k-1}$ and $R_{k}$, respectively. In Step 3, $P_{k/k-1}^{-1/2}$ and $R_{k}^{-1/2}$ are inverse matrixes of $P_{k/k-1}^{1/2}$ and $R_{k}^{1/2}$, respectively. The Huber function is a weight function of the residual, which reduces the weight of the measurement with large error.

Navigation state errors are obtained after each filter update and then used to correct the current navigation state. The process is as follows:

(33) $$L^{+}=\hat{L}-\frac{\delta p_{N}}{R_{M}+\hat{h}}$$

(34) $$\lambda^{+}=\hat{\lambda }-\frac{\delta p_{E}}{\left(R_{N}+\hat{h}\right)\cos\hat{L}}$$

(35) $$h^{+}=\hat{h}+\delta p_{D}$$

(36) $$v^{n+}={\hat{v}}^{n}-\delta v^{n}$$

(37) $$a^{n+}={\hat{a}}^{n}-\delta a^{n}$$

(38) $$b_{c}^{+}={\hat{b}}_{c}+\delta b_{c}$$

(39) $$d_{c}^{+}={\hat{d}}_{c}+\delta d_{c}$$

Process noise variance matrix Q is usually a diagonal matrix:

(40) $$Q=\left[\begin{array}{ccccc} Q_{p} & 0 & 0 & 0 & 0\\ 0 & Q_{v} & 0 & 0 & 0\\ 0 & 0 & Q_{a} & 0 & 0\\ 0 & 0 & 0 & Q_{\mathrm{bc}} & 0\\ 0 & 0 & 0 & 0 & Q_{\mathrm{dc}} \end{array}\right]$$

$Q_{p}$,$Q_{v}$, and $Q_{\mathrm{bc}}$ are unimportant parameters that can be set to zero. $Q_{a}$ and $Q_{\mathrm{dc}}$ are the key parameters that affect the performance of the navigation filter and that need to be carefully considered. The magnitudes of $Q_{a}$ and $Q_{\mathrm{dc}}$ depend on the maximal dynamic of the vehicle and the quality of the oscillator, respectively. Their values are generally set using a rule of thumb that the higher the vehicle’s dynamic is, the larger the value of $Q_{a}$ and the poorer the oscillator’s quality is, the larger the value of $Q_{\mathrm{dc}}$.

The measurement noise variance of each satellite is related to the intensity of the received signal. In our study, pseudorange noise variance is modeled as

(41) $$\sigma_{\rho }^{2}=\lambda_{\mathrm{co}}^{2}\frac{A_{\rho }}{nT_{\mathrm{coh}}C/N_{0}}$$

where n is the number of average points in Equation (8), $C/N_{0}$ is the carrier-to-noise ratio (CNR), $A_{\rho }$ is a coefficient whose value is related to the autocorrelation function of the pseudo-code and the code phase discriminator model, which can be obtained by numerical simulation. The pseudorange rate noise variance is modeled as

(42) $$\sigma_{\dot{\rho }}^{2}=\lambda_{\mathrm{ca}}^{2}\frac{{\left(A_{\dot{\rho }}B_{n}\right)}^{3}}{C/N_{0}}$$

where $A_{\dot{\rho }}$ is the coefficient for calculating the pseudorange rate noise variance, and it is also obtained by numerical simulation.

### 2.3. Weak Signal Channel Strategy

Although a third-order PLL can track high-dynamic signals, it is vulnerable. Because there are two integrators in the loop filter, the integral value is easy to diverge in the case of large phase discriminator noise. In practical tests, even a few seconds of a weak signal can cause third-order PLL loss of lock, but it cannot recover on its own ability when the signal becomes stronger. To ensure the continuity of signal tracking, it is necessary to design an additional reacquisition module when third-order PLL is used in a scalar receiver. In vector tracking, the fast recovery of third-order PLL is easy because of the superiority of the control strategy.

When the CNR of a channel is less than a preset threshold, the signal is judged as weak. In this case, $f_{\mathrm{ca}}$ and ${\dot{f}}_{\mathrm{ca}}$ in the pseudorange rate measurement module may be diverging. Therefore, the recovery of third-order PLL includes two aspects: Carrier frequency recovery and carrier frequency change rate recovery. The former is simple. Because $f_{\mathrm{ca}}^{\mathrm{NCO}}$ is corrected by the receiver’s velocity, it is supposed to be accurate when the navigation filter is working properly. So $f_{\mathrm{ca}}^{\mathrm{NCO}}$ is assigned to $f_{\mathrm{ca}}$ after each navigation filter update. The latter requires some additional calculations. First, the calculation formula of carrier frequency change rate is derived. The relative velocity between receiver and satellite is expressed as

(43) $$v_{\mathrm{los}}=\left(v_{s}-v_{p}\right)\cdot \left(r_{s}-r_{p}\right)\frac{1}{R}$$

where the subscript s denotes the satellite, subscript p denotes the receiver, r denotes the position vector, v denotes the velocity vector, and $R=\left\|r_{s}-r_{p}\right\|$. By taking the derivative of both sides of Equation (43) and considering $\dot{R}=v_{\mathrm{los}}$, the relative acceleration between receiver and satellite can be obtained by

(44) $$a_{\mathrm{los}}=\frac{a_{\mathrm{ps}}\cdot r_{\mathrm{ps}}}{R}+\frac{v_{\mathrm{ps}}\cdot v_{\mathrm{ps}}}{R}-\frac{{\left(v_{\mathrm{ps}}\cdot r_{\mathrm{ps}}\right)}^{2}}{R^{3}}$$

where a denotes the acceleration vector, $a_{\mathrm{ps}}=a_{s}-a_{p}$, $v_{\mathrm{ps}}=v_{s}-v_{p}$, $r_{\mathrm{ps}}=r_{s}-r_{p}$. The relative acceleration is related to the velocity and acceleration of the satellite and receiver. The satellite’s velocity and acceleration can be calculated by ephemeris [23,24]. The receiver’s velocity is obtained by solving a velocity observation equation. The receiver’s acceleration comes from the navigation state in Equation (25), but it is with respect to the geographic frame. Acceleration with respect to Earth-centered Earth-fixed (ECEF) frame $a_{p}$ is calculated with the Coriolis theorem:

(45) $$a_{p}=C_{n}^{e}\left(a^{n}+\omega_{\mathrm{en}}^{n}\times v^{n}\right)$$

where $C_{n}^{e}$ is the transformation matrix of the geographic frame to the ECEF frame, $\omega_{\mathrm{en}}^{n}$ is the angular velocity between the two frames. Then ${\dot{f}}_{\mathrm{ca}}$ can be calculated by $a_{\mathrm{los}}$ considering receiver clock drift:

(46) $${\dot{f}}_{\mathrm{ca}}=-\frac{a_{\mathrm{los}}}{\lambda_{\mathrm{ca}}\left(1+df_{r}\right)}$$

Under the recovery action, the carrier phase tracking of weak signal degenerates into a first-order PLL, and the proportional term of carrier phase error $K_{1}\delta \theta$ is used to correct the carrier phase. For weak signals, because δθ has a large error, phase locking is not guaranteed, and using $K_{1}\delta \theta$ correction is only an attempt. When the signal becomes stronger, due to the small error of $f_{\mathrm{ca}}$ and ${\dot{f}}_{\mathrm{ca}}$, the carrier phase relocks, and the third-order PLL is restored. In this control mode, the recovery of an interrupted signal only needs a very short transition time, which is the phase locking time of a first-order PLL. The disadvantage that third-order PLL is an easy loss of lock is overcome.

### 2.4. Implementation of Hardware Platform

#### 2.4.1. Structure of Hardware Platform

**Figure 5:**
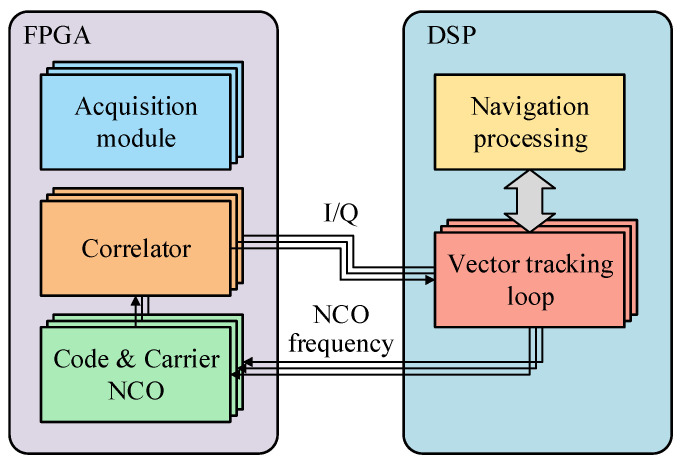
Functional diagram of vector tracking hardware platform.

The vector tracking verification hardware platform used in this paper is an FPGA + DSP architecture. The functional diagram of the platform is shown in Figure 5. The FPGA is Intel’s Cyclone V 5CEFA9F23I7, and the DSP is TI’s TMS320C6748. The DSP scans the state of all channels every 0.505 ms. If a channel completes a correlation operation, the FPGA outputs the channel’s *I*/*Q* correlation value. The vector tracking loop calculates the NCO frequency of each channel according to *I*/*Q* correlation values. Every 50 ms, all channels latch the current code phase, and FPGA generates an interrupt signal to trigger navigation processing in the DSP.

#### 2.4.2. Code Phase Correction

**Figure 6:**
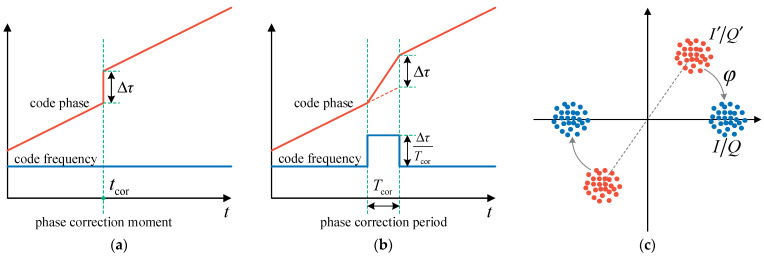
(**a**) Direct code phase correction; (**b**) indirect code phase correction; (**c**) carrier phase correction.

Intuitively, code phase correction is to assign a desired phase or phase correction value to the code NCO at the correction moment, as shown in Figure 6a. However, the code phase of the code NCO cannot be directly controlled in practice. An indirect correction method is required. A phase correction period is set, typically 1 ms. In this period, code NCO frequency adds a phase correction frequency based on the normal value. At the end of the phase correction period, the code phase error is corrected, as shown in Figure 6b.

#### 2.4.3. Carrier Phase Correction

In practice, it is not necessary for the local replica carrier to be in phase with the actual carrier for signal tracking. Frequency matching is the only requirement. On the one hand, the incoherent code phase discriminator does not require carrier phase locking. On the other hand, the carrier phase error can be corrected after coherent integration. As shown in Figure 6c, when the phase error between the local and actual carrier is φ, the distribution of *I*/*Q* correlation values is orange dots. *I*/*Q* correlation values can be corrected to a zero phase by a rotation transformation of Equation (47). This is equivalent to phase locking.

(47) $$\{\begin{array}{l} I=I^{\prime }\cos\varphi +Q^{\prime }\sin\varphi \\ Q=-I^{\prime }\sin\varphi +Q^{\prime }\cos\varphi \end{array}$$

Thus, in receiver design, each channel has a variable used to record the current carrier phase error. The six *I*/*Q* outputs first perform Equation (47), and then perform the code and carrier phase discriminator algorithm. Carrier phase correction can be realized by modifying the carrier phase error variable after each PLL update.

#### 2.4.4. Time Control

There are three basic time periods in VTL, which have been mentioned many times before. They are summarized in Table 1.

#### 2.4.5. Pseudorange Rate Measurement Synchronization

**Figure 7:**
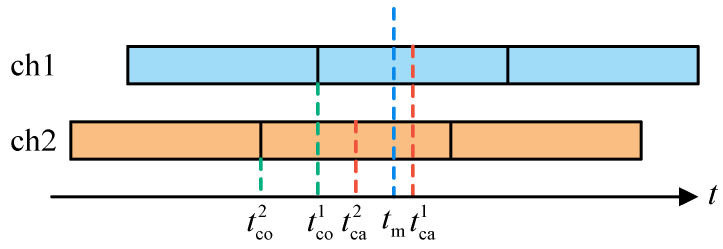
Diagram of pseudorange rate measurement process.

For the pseudorange measurement, all channels simultaneously latch the current code phases at the positioning moment, and they are synchronous. However, because the carrier frequency estimator update moment aligns with the end of coherent integration, all channels are asynchronous. Special processing is required to obtain synchronous pseudorange rate measurements. Figure 7 illustrates the process of pseudorange rate measurement. $t_{\mathrm{co}}^{1}$ and $t_{\mathrm{co}}^{2}$ are the end time of a pseudo-code period, $t_{\mathrm{ca}}^{1}$ and $t_{\mathrm{ca}}^{2}$ are the middle time of the next pseudo-code period, and $t_{m}$ is the pseudorange rate measurement moment. In fact, the estimated carrier frequency at $t_{\mathrm{co}}$ is the carrier frequency at $t_{\mathrm{ca}}$. Time difference $t_{m}-t_{\mathrm{ca}}$ needs to be considered to obtain the pseudorange rate measurements for all channels at $t_{m}$. The precise carrier frequency at $t_{m}$ can be calculated by extrapolation using carrier frequency change rate:

(48) $$f_{\mathrm{ca}}^{m}=f_{\mathrm{ca}}+{\dot{f}}_{\mathrm{ca}}\left(t_{m}-t_{\mathrm{ca}}\right)=f_{\mathrm{ca}}+{\dot{f}}_{\mathrm{ca}}\left[\left(t_{m}-t_{\mathrm{co}}\right)-\left(t_{\mathrm{ca}}-t_{\mathrm{co}}\right)\right]$$

where $t_{m}-t_{\mathrm{co}}$ is obtained by the code phase at $t_{m}$, and $t_{\mathrm{ca}}-t_{\mathrm{co}}$ is equal to half of a pseudo-code period.

#### 2.4.6. Workflow of Vector Receiver

The workflow of a vector receiver can be divided into four stages. They are described in Table 2.

## 3. Experiments and Results

GNSS signal simulator is efficient equipment to test a GNSS receiver, which can accurately and repeatedly generate GNSS signals received by a custom motion vehicle in a laboratory. In our study, a Spirent GSS7700 simulator was used to generate high-dynamic GPS L1 C/A signals for experiments. To create the desired motion scenario, a sequence containing a timestamp, position, and velocity was generated by self-developed trajectory generator software and then converted into a user motion file with a umt extension. SimGEN software can process the file to simulate GPS signals. In our experiments, two high-dynamic scenarios were set up to verify the performance of VTL, an ultrahigh-dynamic scenario, and a normal high-dynamic scenario. Each scenario was tested with STL and VTL for comparison. To simply compare the performance of loop structures, the common parameters for STL and VTL were set the same. The detailed parameters are listed in Table 3.

### 3.1. Ultrahigh-Dynamic Scenario Experiment

**Figure 8:**
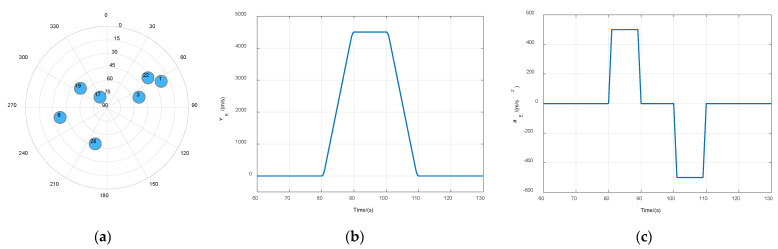
(**a**) Satellite constellation of experiment scenario; (**b**) curve of eastward velocity; (**c**) curve of eastward acceleration.

**Figure 9:**
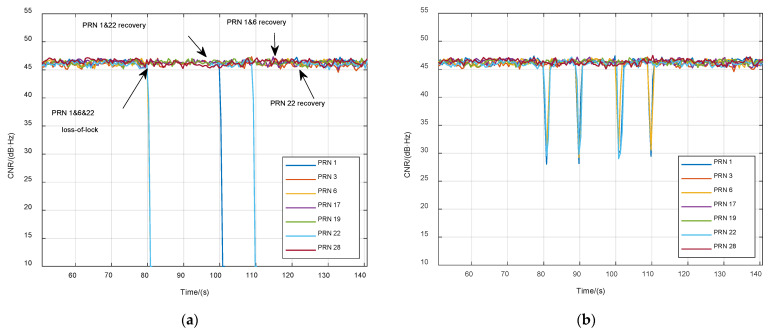
Carrier-to-noise ratio (CNR) of tracking satellites in ultrahigh-dynamic scenario. (**a**) Scalar tracking loop (STL); (**b**) vector tracking loop (VTL).

The ultrahigh-dynamic scenario was an eastward accelerated trajectory in which the maximal acceleration was 50 g, the jerk was 50 g/s, and the maximal velocity was 4500 m/s. The satellite constellation, and the curves of velocity and acceleration of the trajectory are shown in Figure 8. Results of the two different tracking methods are shown in Figure 9. No.1, 6, and 22 satellites had large acceleration relative to the receiver, due to the lower elevating angle during the acceleration period. The carrier dynamic pressure of these signals exceeded the tracking threshold of the third-order PLL in STL, resulting in loss of lock at 80 s, 100 s, and 110 s, as shown in Figure 9a. Loss-of-lock signals need several seconds to recover. In VTL, the CNR of those three signals dropped only for two seconds at the moment of a sudden jerk because the four low-dynamic signals could maintain the operation of the navigation filter, and the correct navigation state could provide accurate NCO control command for the high-dynamic channels to ensure effective tracking. Thus, the VTL showed better performance in high-dynamic signal tracking.

### 3.2. Normal High-Dynamic Scenario Experiment

**Figure 10:**
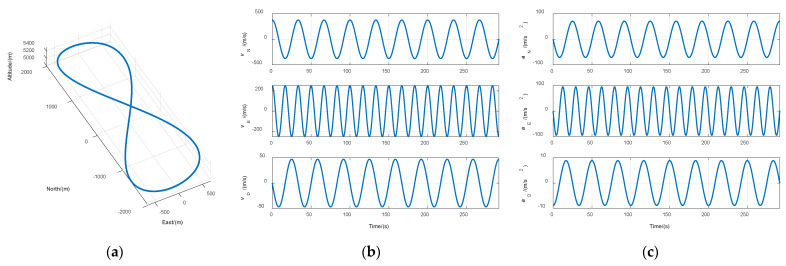
Simulated trajectory curves. (**a**) 3D position; (**b**) velocity; (**c**) acceleration.

The normal high-dynamic scenario was a figure-eight trajectory [25]. Trajectory length was 10,000 m, the average speed was 300 m/s, and altitude variation was 500 m. The curves of the 3D position, velocity, and acceleration of the trajectory are shown in Figure 10. This experiment contains three cases: Constant signal power, signal attenuation, and partial signal outage.

**Figure 11:**
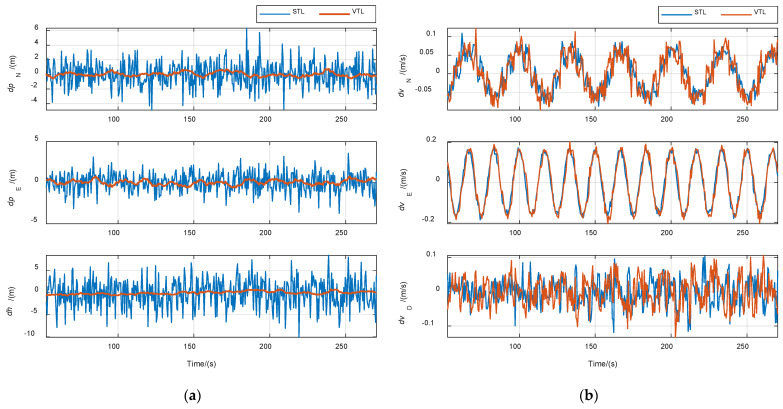
Comparison of navigation errors between STL and VTL in case of constant signal power. (**a**) Position error; (**b**) velocity error.

The purpose of the first case is to verify the improvement of VTL in navigation accuracy. The CNR of all signals was set to 46 dB·Hz. Results are shown in Figure 11. The position and velocity solution were both performed by single point mode in STL and VTL without other processes. Navigation root-mean-square (RMS) errors are listed in Table 4. The position error of VTL was obviously smaller than that of STL. The position error also reflected the tracking accuracy of the local replica code. Therefore, the code tracking accuracy of VTL was higher than that of STL. Theoretically, the same position accuracy could be obtained when the position output of STL passed the same navigation filter as that of VTL, but the code phase of the NCO could not be changed in STL. The velocity errors of the two methods were almost the same, because the navigation filter’s bandwidth was set to be relatively wide for tracking high-dynamic signals, resulting in no filtering effect on the velocity solution. Thus, the carrier frequency tracking accuracy of VTL cannot be improved unless the navigation filter’s bandwidth is compressed, or an inertial measurement unit (IMU) is used.

**Figure 12:**
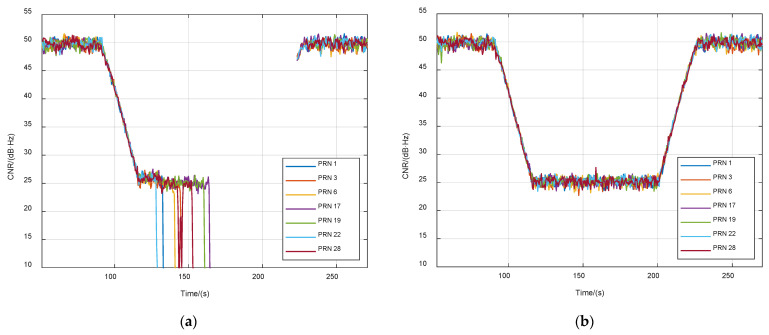
CNR of tracking satellites in case of signal attenuation. (**a**) STL; (**b**) VTL.

**Figure 13:**
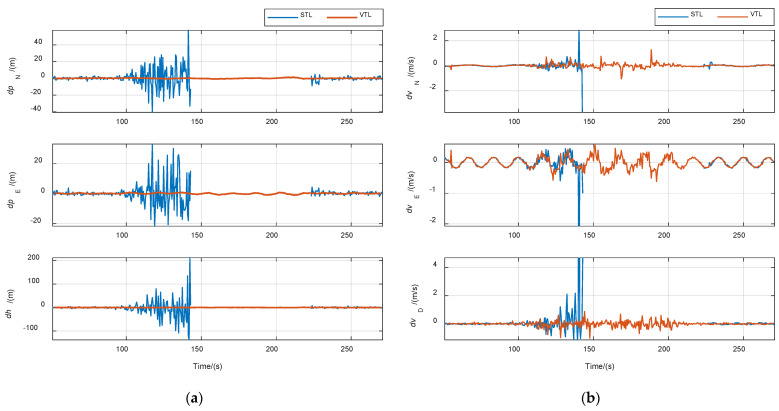
Comparison of navigation errors between STL and VTL in case of signal attenuation. (**a**) Position error; (**b**) velocity error.

The purpose of the second case was to verify the weak-signal-tracking performance of VTL. The initial CNR of all signals was set to 50 dB·Hz. Then the signal power was reduced at a rate of 1 dB/s from 90 s. From 200 s, the power increased at a rate of 1 dB/s until 50 dB·Hz. Results are shown in Figure 12 and Figure 13. When the CNR dropped to 25 dB·Hz, STL channels lost the lock one after another, but all VTL channels kept tracking. During the weak signal period, the STL could not output navigation results. On the contrary, the VTL still provided relatively accurate navigation output, because it made use of the power of all channels. Thus, the tracking sensitivity of VTL was higher.

**Figure 14:**
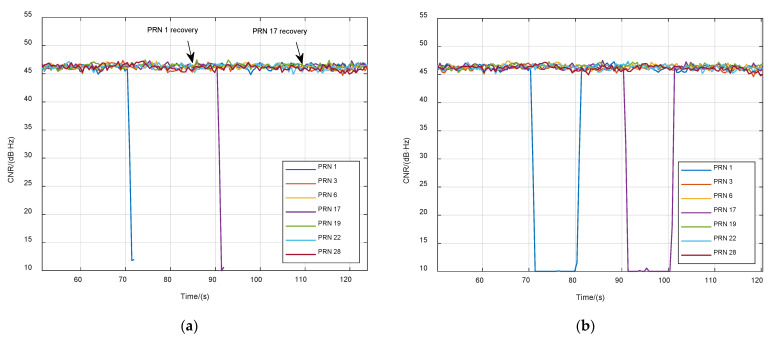
CNR of tracking satellites in case of a partial signal outage. (**a**) STL; (**b**) VTL.

The third case demonstrates the ability of VTL to quickly recover interrupted signals. The signals of PRN 1 and PRN 17 were closed for 10 s at 70 s and 90 s, respectively. Figure 14 shows the different results of STL and VTL. Signals were immediately relocked in VTL when signal power was recovered. In STL, the reacquisition process took longer.

### 3.3. Calculation Time Statistics

Because the calculation of the code phase and carrier NCO frequency correction value, and the pseudorange and pseudorange rate measurement contain only a few simple arithmetic operations, the computation load of VTL is mainly on the navigation processing, in particular the navigation filter. To verify the real-time performance of VTL, the average calculation time of one instance of navigation processing was counted in cases of different numbers of tracking satellites. DSP frequency was 456 MHz. The statistical results are shown in Table 5. A cubic polynomial can be used to fit the number of satellites and calculation time. When the number of satellites processed by the navigation filter was 12, the proposed vector tracking method met the real-time requirement. Because NCO control takes almost no time, more satellites can be tracked in VTL; only a satellite selection algorithm is required to limit the number of satellites used by the navigation filter.

## 4. Discussion

Although the study of VTL in this paper is based on GPS L1 C/A signal, which is a legacy signal using BPSK (binary phase shift keying) modulation, the VTL can also be used to track BOC (binary offset carrier) modulated signal, which is the new signal transmitted by modern GNSS satellites, such as GPS L1C signal, BDS B1C signal, and Galileo E1 signal. In BOC modulation, pseudo-code chips are multiplied by sine square wave subcarriers to realize the nature of split-spectrum [26]. In essence, the structure of the BOC signal is still code and carrier. Therefore, the proposed VTL framework is applicable. Only the code generator, code phase discriminator, and carrier phase discriminator must be modified to adapt to the new signal.

In addition, the VTL has a potential in an antenna array-based GNSS receiver, which is used to mitigate RF interference. When using the minimum mean squared error (MMSE) algorithm to realize beamforming in the post-correlation technique, a reference signal is necessary [27]. The VTL can provide this reference signal more precisely.

At present, the receiver hardware platform still adopts the architecture of separated components, and DSP and FPGA chips are larger and consume more power. To further promote the practical application of vector tracking technology, the program will be transplanted to an SoC-based receiver platform, and an IMU will be introduced to assist.

## 5. Conclusions

Instead of using code phase and carrier frequency to directly control NCO, the vector tracking framework proposed in this paper calculates code phase and carrier frequency errors for incremental correction. This approach makes NCO control more flexible, so that VTL can be easily implemented based on existing receivers without much modification. A hardware receiver based on the proposed framework was realized on an FPGA + DSP platform with good real-time performance. Through effective information exchange between channels, the vector receiver had a stronger tracking ability of high-dynamic signals. In addition, the advantages of tracking sensitivity and bridging interrupted signals were verified.

## Figures and Tables

**Table 1:** Summary of three basic time periods in VTL.

Symbol	Meaning	Description
$T_{\mathrm{coh}}$	Coherent integration time	This depends on signal intensity. The weaker the signal is, the longer the coherent integration time.
$T_{\mathrm{NCO}}$	Code and carrier NCO update period	For better tracking of high-dynamic signals, this period needs to be as small as possible, even if the coherent integration time is long. This is usually equal to the minimal integration time of the correlator, which was 1 ms on our hardware platform.
$T_{\mathrm{nav}}$	Navigation update period	Theoretically, the faster the navigation update, the better. But fast navigation update has the problem of real-time computation. So, there is a trade-off to be made. Besides that, this period also depends on the hardware design. This was 50 ms on our hardware platform.

**Table 2:** Workflow of vector receiver.

Stage	Description
Before vector tracking	The receiver starts in scalar tracking mode and tracks satellite signals using conventional acquisition and tracking methods.
Enter vector tracking	When the number of satellites that are tracking and have ephemeris is greater than or equal to 4, the receiver enters vector tracking mode. The channels that are tracking signals are switched to use VTL.
Vector tracking running	Continuously acquire untracked satellites. When a new satellite is tracked, it is switched to use VTL.
Exit vector tracking	When the number of effective satellites (such as the CNR is larger than 23 dB·Hz) is less than 4 for a period of time, the navigation filter becomes untrusted, and the receiver exits vector tracking mode and returns to scalar tracking for a new working cycle.

**Table 3:** Receiver parameters configuration.

	Parameter	Value	Unit
STL	Bandwidth of 2nd-order DLL	2	Hz
	Bandwidth of 3rd-order PLL	18	Hz
	Coherent integration time *	1/5/20	ms
	Loss-of-lock threshold	18	dB·Hz
VTL	Bandwidth of 3rd-order carrier frequency estimator	18	Hz
	Coherent integration time *	1/5/20	ms
	Navigation update period	50	ms
	Loss-of-lock threshold	18	dB·Hz
	$Q_{p}$	0	m^2^
	$Q_{v}$	0	(m/s)^2^
	$Q_{a}$	100^2^	(m/s^2^)^2^
	$Q_{\mathrm{bc}}$	0	m^2^
	$Q_{\mathrm{dc}}$ ^†^	0.3^2^	(m/s)^2^

* The coherent integration time is adjusted according to CNR, 1 ms for larger than 37 dB·Hz, 5 ms for between 37 and 30 dB·Hz, 20 ms for less than 30 dB·Hz in the experiment. ^†^ The oscillator used in the receiver is TCXO, whose stability is poor, so *Q*_dc_ is set to a relatively large value.

**Table 4:** Navigation root-mean-square errors statistics table.

	$dp_{N}/(m)$	$dp_{E}/(m)$	dh/(m)	$dv_{N}/(m/s)$	$dv_{E}/(m/s)$	$dv_{D}/(m/s)$
STL	1.68	1.17	3.21	0.047	0.115	0.039
VTL	0.28	0.29	0.34	0.051	0.120	0.042

**Table 5:** The average calculation time of one instance of navigation processing.

Number of Satellites	Average Calculation Time/(ms)
4	1.3
6	2.6
8	4.7
10	8
12	12.8

## Data Availability

The data presented in this study are available on request from the corresponding author.
